# Sulforaphane Attenuates Gentamicin-Induced Nephrotoxicity: Role of Mitochondrial Protection

**DOI:** 10.1155/2013/135314

**Published:** 2013-04-04

**Authors:** Mario Negrette-Guzmán, Sara Huerta-Yepez, Omar Noel Medina-Campos, Zyanya Lucía Zatarain-Barrón, Rogelio Hernández-Pando, Ismael Torres, Edilia Tapia, José Pedraza-Chaverri

**Affiliations:** ^1^Departamento de Biología, Facultad de Química, UNAM, 04510 Mexico City, DF, Mexico; ^2^Unidad de Investigación en Enfermedades Oncológicas, Hospital Infantil de México “Federico Gómez”, 06720 Mexico City, DF, Mexico; ^3^Sección de Patología Experimental, Instituto Nacional de Ciencias Médicas y Nutrición “Salvador Zubirán”, 14000 Mexico City, DF, Mexico; ^4^Unidad del Bioterio, Facultad de Medicina, UNAM, 04510 Mexico City, DF, Mexico; ^5^Laboratorio de Fisiopatología Renal, Departamento de Nefrología, Instituto Nacional de Cardiología “Ignacio Chávez”, 14080 Mexico City, DF, Mexico

## Abstract

Sulforaphane (SFN), an isothiocyanate naturally occurring in Cruciferae, induces cytoprotection in several tissues. Its protective effect has been associated with its ability to induce cytoprotective enzymes through an Nrf2-dependent pathway. Gentamicin (GM) is a widely used antibiotic; nephrotoxicity is the main side effect of this compound. In this study, it was investigated if SFN is able to induce protection against GM-induced nephropathy both in renal epithelial LLC-PK1 cells in culture and in rats. SFN prevented GM-induced death and loss of mitochondrial membrane potential in LLC-PK1 cells. In addition, it attenuated GM-induced renal injury (proteinuria, increases in serum creatinine, in blood urea nitrogen, and in urinary excretion on N-acetyl-**β**-D-glucosaminidase, and decrease in creatinine clearance and in plasma glutathione peroxidase activity) and necrosis and apoptosis in rats. The apoptotic death was associated with enhanced active caspase-9. Caspase-8 was unchanged in all the studied groups. In addition, SFN was able to prevent GM-induced protein nitration and decrease in the activity of antioxidant enzymes catalase and glutathione peroxidase in renal cortex. In conclusion, the protective effect of SFN against GM-induced acute kidney injury could be associated with the preservation in mitochondrial function that would prevent the intrinsic apoptosis and nitrosative stress.

## 1. Introduction

Sulforaphane (SFN) is an extraordinary potent isothiocyanate naturally occurring in Cruciferae, where it is produced by the action of the enzyme myrosinase on the glucosinolate glucoraphanin [1]. The relevance of SFN in human health has been evident with the numerous studies performed in different human populations. It has evaluated safety, metabolism, and efficacy of interventions with sprouts and extracts of broccoli as vehicle for SFN or glucoraphanin (bacterial microflora of human gastrointestinal tract owns myrosinase activity) in healthy subjects as well as other models of human sickness [2]. In recent years, SFN has attracted much attention as a natural inductor of phase II enzymes in humans and animals. It has been proposed that the induction of the nuclear translocation of Nrf2 and its binding to the antioxidant response element (ARE), whereby the cytoprotective genes transcription is activated, may occur either by disruption of NF-E2 related factor 2 (Nrf2)-Kelch-like ECH-associated protein 1 (Keap 1) interactions or by mitogen-activated protein kinases (MAPK) pathways activation [1, 3]. Accordingly, SFN falls in the category called indirect antioxidants that may or may not be redox active [4], but it is known that the scavenging ability of SFN for several reactive oxygen species (ROS) is very low or negligible [5]. Interestingly, in several cytoprotective effects associated with SFN, a preservation of mitochondrial function has been found [5–12]. Guerrero-Beltrán and coworkers observed that SFN was able to prevent cisplatin-induced mitochondrial membrane potential (MMP) disruption in LLC-PK1 cells and mitochondrial permeability transition pore opening as well as other mitochondrial alterations and nephrotoxicity in rats.

On the other hand, gentamicin (GM) is a widely used worldwide aminoglycoside (AG) antibiotic in the treatment of severe infections caused by Gram-negative, mycobacteria and enterococci. This is mainly due to the typical characteristics of AGs: powerful bactericidal activity, postantibiotic effects, chemical stability, synergy with betalactam antibiotics, low cost and low levels of resistance, and because they are an effective alternative against germs insensitive to other antibiotics. However, nephrotoxicity is the principal limitation of GM therapeutic efficacy [13, 14]. Several recent intrahospital observational studies evidence an incidence of GM-induced acute kidney injury (defined by different criteria) ranging from 20% to 35.6% [15–17]. The molecular and pathophysiological mechanisms of GM-induced nephrotoxicity are well characterized. GM is internalized through the giant endocytic complex that is preferentially expressed in the renal proximal tubular segments S_1_ and S_2_. In these cells, GM is mainly accumulated in lysosomes, the Golgi, and endoplasmic reticulum, producing lysosomal phospholipidosis, unfolded protein response, and other effects, therefore turning on apoptotic and necrotic death pathways [13, 14]. Nevertheless, mitochondria could be a much more sensitive target for GM. In cell culture, it has been found that GM decreases MMP, releases cytochrome c, and induces activation of caspases 9 and 3, which are elements belonging to the events cascade leading to intrinsic apoptosis [18]. In addition, induction of opening of the permeability transition pore in mitochondria isolated from kidneys of rats treated with GM was observed [19].

Because of the emergent importance of mitochondria in the renal toxicity induced by GM, it has been claimed that all of strategies that tend to preserve the normal kidneys performance in patients under an AG treatment scheme will require protection of renal mitochondria [20]. Considering all of the SFN qualities and knowing the nephrotoxicity mechanism of GM, we hypothesized that SFN is able to prevent GM-induced nephrotoxicity. The aim of the present study was to evaluate whether SFN induces a cytoprotective effect on a model of GM-induced acute kidney injury and cultured cells LLC-PK1 treated with GM.

## 2. Materials and Methods

### 2.1. Chemicals and Reagents

SFN (Cat. no. S8044, batch 26817812) was obtained from LKT Laboratories (St. Paul, MN, USA). GM (Garamicina G.U. 160 mg/2 mL, batches 0DPDA006 and 1DPDA002) was purchased from Schering-Plough (México, DF). Dulbecco's Modified Eagle Medium (DMEM), fetal bovine serum (FBS), trypsin, antibiotic (10,000 U/mL penicillin and 10,000 *μ*g/mL streptomycin), 0.4% trypan blue solution, and other tissue culture reagents were purchased from Gibco (México, DF). Cell culture plates were from Nunc (Roskilde, Denmark). 3-(4,5-dimethylthiazol-2-yl)-2,5-diphenyltetrazolium bromide (MTT), dimethylsulfoxide (DMSO), paraformaldehyde, triton X-100, bovine serum albumin (BSA), *p*-nitrophenyl-*N*-acetyl-*β*,D-glucosaminide, nicotinamide adenine dinucleotide reduced form (NADPH), glutathione reductase (GR), and glutathione (GSH) were purchased from Sigma-Aldrich (St. Louis, MO, USA). Trichloroacetic acid (TCA) and hydrogen peroxide (H_2_O_2_) were obtained from J. T. Baker (Xalostoc, Edo. de México, México). Vybrant Apoptosis Assay Kit # 3, containing annexin V conjugated to fluorescein (annexin V-FITC) and propidium iodide (PI), and 5,5′,6,6′-tetrachloro-1,1′,3,3′-tetraethylbenzimidazolyl-carbocyanine iodide (JC-1) were obtained from Molecular Probes, Inc. (Eugene, OR, USA). Mouse monoclonal anti-nitrotyrosine (3-NT) antibody (Cat. no. 189542) was purchased from Cayman Chemical (Ann Arbor, MI, USA). Mouse IgG2a *κ* isotype control (Cat. no. 550339) was purchased from BD Biosciences (San Jose, CA, USA). Rabbit polyclonal anti-caspase 8 antibody (Cat. no. ab4052) was purchased from Abcam Inc. (Cambridge, MA, USA). Goat polyclonal anti-cleaved caspase 9 p10 (h331) antibody (Cat. no. sc-22182), donkey anti-mouse IgG-B biotin-conjugated secondary antibody (Cat. no. sc-2098), rabbit anti-goat IgG-B biotin-conjugated secondary antibody (Cat. no. sc-2774), goat anti-rabbit IgG-B biotin-conjugated secondary antibody (Cat. no. sc-2040), normal goat IgG (Cat. no. sc-2028), and normal rabbit IgG (Cat. no. sc-2027) were obtained from Santa Cruz Biotechnology Inc. (Santa Cruz, CA, USA). *In Situ* Cell Death Detection Kit, POD (Cat. No. 11 684 817 910), was obtained from Roche Applied Science (Mannheim, Germany). Streptavidin-horseradish peroxidase (HRP) and 3,3′-diaminobenzidine (DAB) were obtained from Dako (Carpintería, CA, USA). All other used reagents and compounds were reactive grade and commercially available.

### 2.2. Cell Culture and Viability

Lily Laboratory Culture Porcine Kidney (LLC-PK1, porcine renal epithelium) cells were obtained from American Type Culture Collection (Rockville, MD, USA). This cell line is an accepted model to study AG toxicity [21–23]. LLC-PK1 cells were maintained in DMEM supplemented with 10% FBS and 1% of antibiotic and cultured under permissive conditions: 37°C and 5% CO_2_ [8]. In order to evaluate the effect of SFN on GM-induced toxicity, cells were seeded at a density of 4 × 10^4^ cells/cm^2^ onto 48-well plates and used for the experiment on the following day. Cells were incubated for 24 h with SFN (1–10 *μ*M) or medium before the GM addition. At the end of preincubation period SFN or culture medium was replaced by fresh SFN or medium, adding 8 mM GM to some cells groups in order to induce toxicity. Every 24 h, along 72 h of GM exposure, the culture medium was replaced by fresh medium with the same previous characteristics [24]. Cell viability was assessed by MTT reduction [8]. At the end of 96 h of experiment, medium was removed and cells were washed twice with phosphate-buffered saline (PBS) pH 7.4. Thereafter, cells were incubated in medium containing MTT (0.125 mg/mL) at 37°C for 1 h in humidified air supplemented with 5% CO_2_. Medium was then discarded, and the formazan crystals deposited in each well bottom were dissolved in 200 *μ*L of 0.1 N HCl in isopropanol. Absorbance was determined at 570 nm using a Synergy HT multimode microplate reader (Biotek Instruments Inc, Winooski, VT, USA). In additional experiments, bright field images were obtained by phase contrast microscopy (Nikon Eclipse TS100F, Nikon Co, Tokyo, Japan) to compare cell morphology in all experimental conditions.

### 2.3. Cell Death Assays

In order to estimate cell death and characterize its phenotypes, LLC-PK1 detached cells in 24-well plates (CT, 5 *μ*M SFN, 8 mM GM and SFN + GM groups) were collected from media, pelleted by centrifugation (1000 ×g, 3 min) and counted in a haemocytometer at 24, 48, and 72 h of GM exposure. Attached cells were also counted at the end of the GM treatment by trypsinization and using the trypan blue exclusion assay. Viable cells were sorted by this technique. Because there was a lot of cell fragmentation (mainly in the GM and SFN + GM groups), only attached cells were incubated in annexin V-FITC and PI probes at the end of the GM treatment [25, 26]. After 15 min of incubation with annexin V-FITC/PI solution, cells were washed with annexin V-binding buffer (10 mM HEPES, 140 mM NaCl, 2.5 mM CaCl_2_, pH 7.4) and photographed in a Nikon Eclipse TS100F inverted microscope provided with G-2A (excitation 510–560 nm and emission 590 nm) and B-2A (excitation 450–490 nm and emission 515 nm) filters. Thereafter cells were trypsinized, centrifuged, resuspended in 100 *μ*L of annexin V-binding buffer, and put in a black microplate and their fluorescence emission was measured at 528 (annexin V-FITC) and 600 (PI) nm in a Synergy HT multimode microplate reader.

### 2.4. MMP Measurements

MMP was assessed by using the cationic lipophilic fluorescence dye JC-1 which forms potential-dependent J-aggregates [27]. The cells were first seeded in 24-well plates and used for the experiment on the following day. Thereafter, cells were incubated for 24 h with 5 *μ*M SFN or medium and then were treated with GM (8 mM), SFN + GM, SFN, or medium. Following the exposure to GM for 1, 3, 6, 12 and 24 h, the medium was replaced by medium containing 10 *μ*M JC-1 and cells were incubated at 37°C for 30 min in the dark. Cells were then rinsed with PBS and examined under an inverted microscope (Nikon, Eclipse TS 100) provided with G-2A filter (excitation 510–560 nm and emission 590 nm) for qualitative studies [8, 18].

### 2.5. Animals

Male Wistar rats with an initial body weight of 230–260 g were used. Animals were maintained under 12 h light/dark cycles at controlled temperature, having *ad libitum* access to water and standard food. This experimental study was approved by the Local Committee for the Care and Use of Laboratory Animals (FQ/CICUAL/038/12) and was conducted according to the guidelines of Mexican Official Norm Guide for the use and care of laboratory animals (NOM-062-ZOO-1999) and for the disposal of biological residues (NOM-087-ECOL-1995).

### 2.6. Experimental Design

Animals were randomly divided into four groups: (i) control group (CT, *n* = 8): the animals were injected subcutaneously (s.c.) with isotonic saline solution (ISS) every 12 h for 4 days, an additional daily injection with ISS (vehicle of SFN) was made intraperitoneally (i.p.) at a time between the previously described injections; (ii) the rats of SFN group (*n* = 8) were administered s.c. with ISS and i.p. with SFN (1.0 mg/kg/24 h) dissolved in ISS; (iii) the rats of the GM group (*n* = 12) were administered s.c. with GM at a dose of 70 mg/kg/12 h instead of ISS every 12 h [28, 29], ISS was injected once every day during the 4 days of treatment; and (iv) rats of the SFN + GM group (*n* = 10) were injected s.c. with 70 mg/kg of GM every 12 h during 4 days and i.p. with SFN 1.0 mg/kg/24 h between the two daily GM injections. On the fourth day of treatment, rats were placed in metabolic cages to collect 24 h urine for the measurement of nephrotoxicity markers (proteinuria and urinary excretion of N-acetyl-*β*-D-glucosaminidase (NAG)). At the end of this period rats were anesthetized with an injection of sodium pentobarbital (60 mg/kg) and blood was obtained via aorta using a syringe containing heparin and a needle no.18 at room temperature. Plasma was separated and stored at −20°C until further markers of renal damage (plasma creatinine, creatinine clearance, blood urea nitrogen (BUN), and activity of glutathione peroxidase (GPx) in plasma) were measured. Right kidney was quickly removed, weighed, divided in *≈*0.3 g slices and frozen on liquid nitrogen, and stored at −80°C. Remaining kidney was perfused with PBS pH 7.4 and 4% formaldehyde solution in PBS, removed, and preserved in formaldehyde solution for histopathological and immunohistochemistry studies.

### 2.7. Analytical Methods

BUN and creatinine in plasma and urine were measured using an autoanalyzer. Creatinine clearance was calculated with the standard formula. Proteinuria was determined by a turbidimetric method using 12.5% TCA and 420 nm as wavelength [30, 31]. Urinary NAG activity was determined measuring the anionic form of *p*-nitrophenol at 405 nm at alkaline pH; this molecule is formed from *p*-nitrophenyl-*N*-acetyl-*β*-D-glucosaminide which is used in the assay as NAG substrate [32].

### 2.8. Histopathological Studies

The kidney fixed in formaldehyde was dehydrated in graded ethylic alcohol and embedded in paraffin. Sections of 3 *μ*m thickness were obtained for staining with hematoxylin-eosin (H&E) and the quantitative histological damage was determined by a Leica QWin Image Analyzer (Cambridge, UK) [30].

### 2.9. Immunohistochemistry and Digital Pathology Analysis

Immunohistochemical staining for caspase-8, cleaved caspase-9, and 3-NT was performed in 3 *μ*m deparaffinized renal tissue slices. Antigens were recuperated by boiling for 20 min in 0.01% sodium citrate solution, pH 6.0. Endogenous peroxidase activity was blocked with 3% H_2_O_2_ solution in methanol for 30 minutes. Antibody nonspecific binding was inhibited by incubation in a 2% solution of normal swine serum in PBS (PBS-NSS) for 60 min. Slides were incubated overnight at room temperature with anti-caspase-8 (1 : 500) anti-cleaved caspase-9 (1 : 1,000), and anti-3-NT (1 : 250) primary antibodies. The following day, slides were washed five times for 8 min in PBS 1x pH 7.4. After washing, slides were incubated for 30 min at room temperature with a second biotinylated antibody (1 : 500) and for 30 min at room temperature with streptavidin conjugated to HRP. For color developing, DAB was used from one to five min. The reaction was stopped with distilled water and the slides were counterstained with hematoxylin. Finally, tissues were dehydrated and fixed with Mount E-2 medium (Shandon Laboratory, Pittsburgh, PA, USA). Slides were scanned in order to obtain electronic files. Immunohistochemical stains were digitally analyzed with Aperio CS (San Diego, CA, USA) digital pathology equipment, and expression density (brown color) in five 10^6^ *μ*m^2^ areas belonging to the renal cortex was evaluated with an algorithm determining pixel density with the program included in the Aperio ScanScope System (San Diego, CA, USA). Active caspase-9 sections were analyzed under a microscope Olympus BX-40 and immunopositives regions were quantified using the software Image-Pro Plus 6.2, Media Cybernetics (Silver Spring, MD, USA). Based on the obtained results, the mean was calculated for each rat and for each group. In order to decrease variability, all the samples in each group were simultaneously processed in one experiment and a single antibody aliquot diluted in PBS-NSS was used.

### 2.10. Apoptosis Detection

DNA fragmentation was evaluated by terminal-deoxynucleotidyltransferase mediated dUTP-digoxigenin nick end labeling (TUNEL) in renal tissue samples. 3 *μ*m sections were subjected to the same immunohistochemical procedure described previously until blocking with PBS-NSS. The enzyme terminal transferase (Tdt) was subsequently added in a 1 : 50 mixture in buffer solution (including fluorescein-conjugated oligonucleotides) to the tissues and incubated for 50 min at 37°C in the dark. Tissues were washed 5 times for 5 min in PBS 1x and subsequently incubated for 30 min with the anti-fluorescein antibody at 37°C. After washing, color was developed by adding DAB and monitored under the light microscope. Slides were scanned and TUNEL-positive nuclei were analyzed with digitalized pathology equipment as described before in the immunohistochemical studies.

### 2.11. Antioxidant Enzymes

Catalase (CAT) activity was assayed in renal cortex by a method based on the disappearance of H_2_O_2_ at 240 nm and glutathione peroxidase (GPx) activity was measured, in blood plasma and renal cortex, by the disappearance of NADPH at 340 nm in a reaction coupled with the enzyme GR [33].

### 2.12. Statistics

Results are expressed as mean ± SEM. Data were analyzed by one-way ANOVA followed by Bonferroni's multiple comparisons test using the software Prism 5.00, GraphPad (San Diego, CA, USA). A *P* value less than 0.05 was considered statistically significant.

## 3. Results

### 3.1. SFN Induces an Increase in MTT Reduction and Prevents the GM-Induced Death in LLC-PK1 Cells

**Figure 1:**
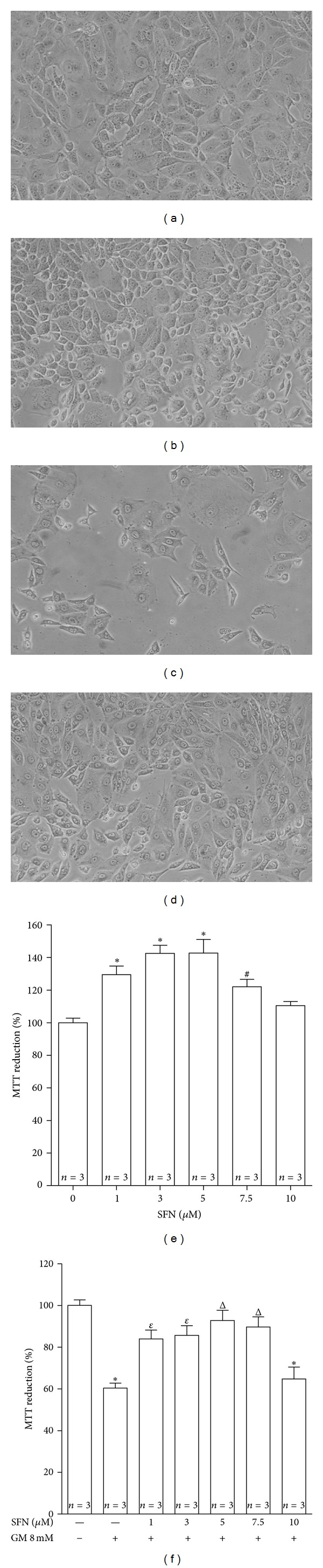
SFN prevents GM-induced death in LLC-PK1 cells. (a–d) Representative images (10x) of LLC-PK1 cells obtained by phase contrast microscopy and (e, f) cell viability (MTT reduction) in the groups under study. A decrease in the number of cells exposed to (c) 8 mM GM for 72 h related to (a) cells without treatment can be observed. This decrease was prevented in (d) cells pretreated with 5 *μ*M SFN for 24 h and coincubated with 5 *μ*M SFN and 8 mM GM for 72 h. (e) Effect of SFN on viability of LLC-PK1 cells. Cells were incubated with 1–10 *μ*M SFN for 96 h. (f) SFN prevents GM-induced cell death. Cells were incubated with 1–10 *μ*M SFN for 24 h and then they were coincubated with 1–10 *μ*M SFN and 8 mM GM for 72 h. Control cells were incubated (e) without SFN or (f) without SFN and GM. Data are mean ± SEM. *P < 0.001, ^#^ *P* < 0.05 versus control cells; ^*ε*^ *P* < 0.01, ^Δ^ *P* < 0.001 versus GM-treated cells.

Figure 1 shows representative phase contrast micrographs and quantification of cell viability by MTT reduction of LLC-PK1 cells under different experimental conditions. Interestingly, an increase in MTT reduction was observed in cells incubated only with 1–7.5 *μ*M SFN. At 5.0 *μ*M SFN, the MTT reduction average was 42.8% greater than that of untreated cells (Figure 1(e)). The 72 h incubation with GM decreased the number of cells (Figure 1(c)) and cell viability (expressed as MTT reduction) to 60.4% (Figure 1(f)). A protective effect by SFN was also found at 1, 3, 5, and 7.5 *μ*M. Even though a clear lineal correlation between viability and SFN concentration was not observed, the protection of SFN was evident with viabilities ranging from 83.9% to 92.8% of the control value (cells without treatment). The maximum increase in MTT reduction of cells coincubated with SFN and GM was observed at 5.0 *μ*M SFN (Figures 1(d) and 1(f)).

**Figure 2:**
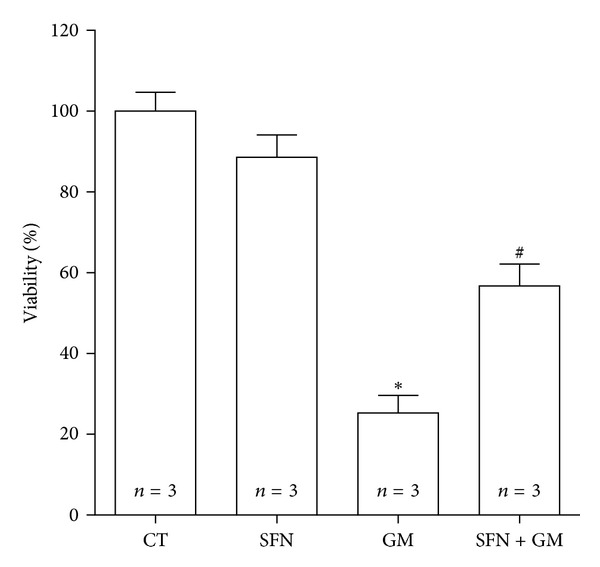
SFN attenuates GM-induced viability drop in LLC-PK1 cells. Cells were incubated with 5 *μ*M SFN for 24 h (SFN and SFN + GM) and then they were co-incubated with 5 *μ*M SFN (SFN and SFN + GM) and 8 mM GM for 72 h (GM and SFN + GM). Control cells (CT) were incubated without SFN and GM. Viability was determined by the trypan blue exclusion assay. Data are mean ± SEM. *P < 0.001 versus CT; ^#^ *P* < 0.05 versus GM.

Table 1 exhibits the number of detached cells for each day of treatment as well as the counting of trypan blue-stained and viable cells from the attached cell fraction. This allows estimating the cell death level in the entire treatment. It should be taken into account that there is an underestimation of death events due to cell fragmentation, which was extensive in GM and SFN + GM groups. However, in this way, more death events could be counted 2.4 times in cells treated with GM than in control cells. It was also observed that the events of death in cells treated with 5 *μ*M SFN and 8 mM GM were approximately 67% of those of GM cells. Viability could be also determined by this means. It is observed that viable cells in the GM group fall until 25% of the control cells while in SFN + GM cells this fall is avoided showing a value of 57% related to CT group (Figure 2). Interestingly, these outcomes were different from those obtained through the MMT reduction method even though the trend in the protective effect is conserved (Figures 1(e) and 1(f)).

### 3.2. SFN Prevents GM-Induced Apoptosis in LLC-PK1 Cells

**Figure 3:**
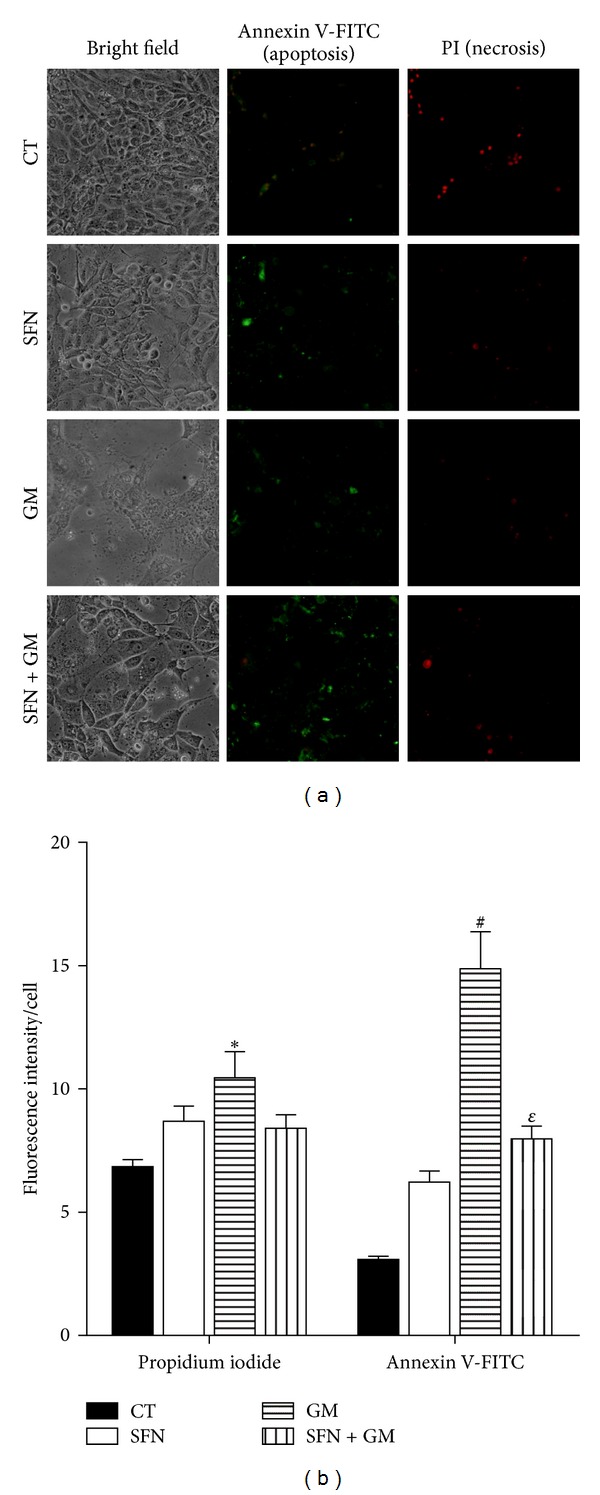
SFN prevents GM-induced apoptosis in LLC-PK1 cells. (a) Representative images (20x) obtained by fluorescence microscopy showing necrotic and apoptotic LLC-PK1 cells after 72 h of exposure to GM. Necrotic cells were stained with PI and apoptotic cells were stained with the annexin V-FITC probe. Cells were incubated with (SFN and SFN + GM groups) or without (CT and GM groups) 5 *μ*M SFN for 24 h, followed by the addition of 8 mM GM (GM), 5 *μ*M SFN (SFN), 5 *μ*M SFN + 8 mM GM (SFN + GM), or medium (CT) for 72 h. All of groups showed similar PI and annexin V-FITC fluorescence, but the cell population was different between them (bright field photographs). (b) Fluorescence intensity corrected for cell number (measured by trypan blue exclusion assay, TBS cells/well + viable cells/well in Table 1) of each group. After pictures taking, fluorescence intensity was measured as was explained in Section 2.3.Data are mean ± SEM of 3 independent experiments. *P < 0.05, ^#^ *P* < 0.001 versus CT; ^*ε*^ *P* < 0.01 versus GM.

Necrosis and apoptosis in LLC-PK1 cells treated with SFN and GM were also estimated using the Vibrant Apoptosis Assay Kit # 3.  Figure 3(a) shows signals due to annexin V-FITC and PI, indicating apoptosis and necrosis, respectively. Apparently, fluorescence intensities and areas are similar in all cell groups however, the difference in cell density for the bright field images of each group should be noticed. In Figure 3(b), it is plotted the fluorescence intensity corrected by the number of cells in each group. Relying on these data, it can be inferred that there was an increase of 1.5-fold in the necrosis and 4.8-fold in apoptosis in cells incubated with GM compared to CT cells. The increase in apoptosis death was significantly attenuated by the treatment with SFN.

### 3.3. SFN Preserves MMP in LLC-PK1 Cells Treated with GM

**Figure 4:**
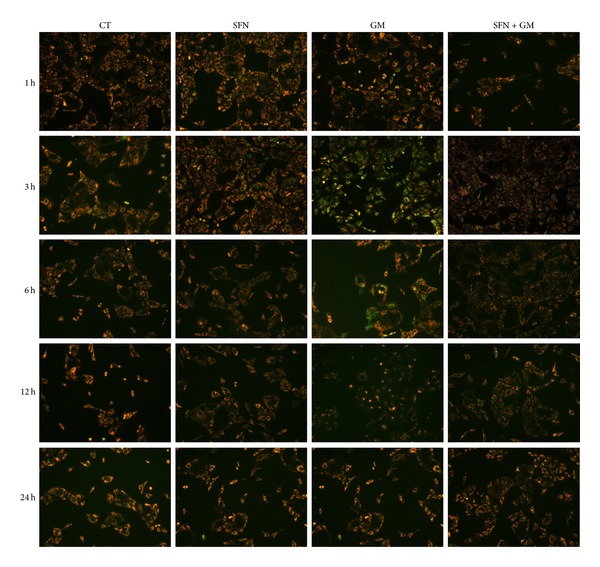
SFN treatment preserves the MMP after the exposure to GM in LLC-PK1 cells. MMP was determined by using the JC-1 probe as an indicator of mitochondrial function. Cells were incubated with (SFN and SFN + GM groups) or without (CT and GM groups) 5 *μ*M SFN for 24 h, followed by the addition of 8 mM GM (GM), 5 *μ*M SFN (SFN), 5 *μ*M SFN + 8 mM GM (SFN + GM), or medium (CT) for 1, 3, 6, 12, or 24 h. Cells with depolarized mitochondria are depicted by green fluorescence (monomeric JC-1) and cells with polarized mitochondria are depicted by orange/yellow fluorescence (aggregate JC-1). Mitochondrial depolarization is observed at 3 and 6 h in cells in the GM group. This fall in MMP is prevented by the SFN treatment. MMP was recovered in the GM groups on 12 h and 24 h of GM exposure.

Mitochondrial depolarization was observed in LLC-PK1 cells after 3 h of GM incubation (green color in the picture, Figure 4). In cells pretreated and coincubated with SFN, the loss of MMP induced by GM was prevented (orange staining in the picture, Figure 4). This trend remained until 6 h. In remarkable way, remaining cells after 12 h of GM exposure (without SFN) seem to recover their MMP but the decrease in the cell number began to be evident.

### 3.4. SFN Treatment Attenuates GM-Induced Renal Dysfunction and Damage in Rats

Neither urinary volume nor body weight was affected by treatment with SFN or GM (Table 2). In contrast, renal weight and right kidney ratio [(kidney weight/body weight) × 100] show an increase in the GM group compared to the CT group (*P* < 0.001), which could not be ameliorated by the SFN treatment (Table 2).

**Figure 5:**
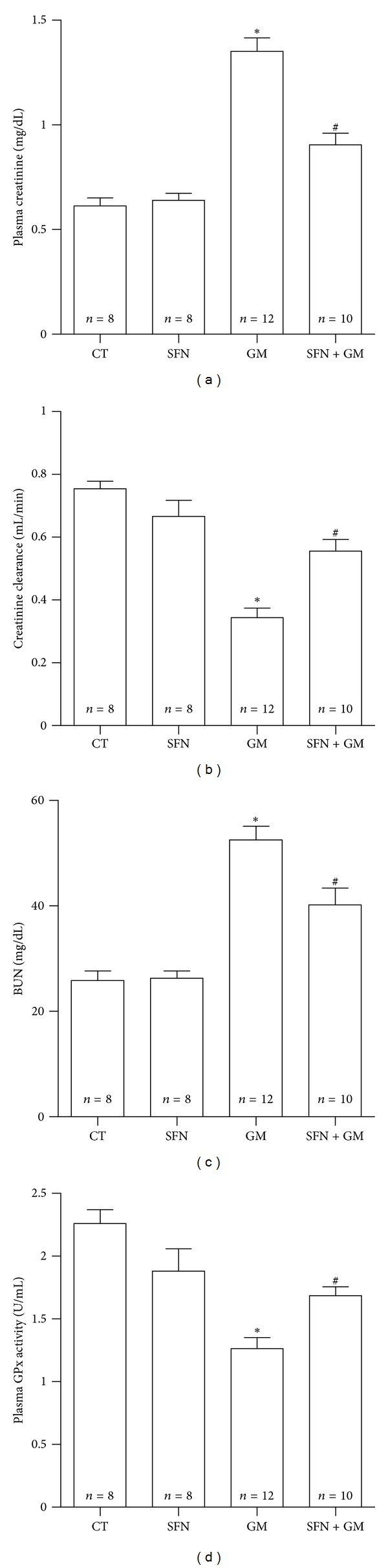
SFN ameliorates GM-induced renal injury in rats. (a) Plasma creatinine, (b) creatinine clearance, (c) BUN, and (d) plasma GPx activity. Data are mean ± SEM. *P < 0.001 versus CT; ^#^ *P* < 0.01 versus GM.

**Figure 6:**
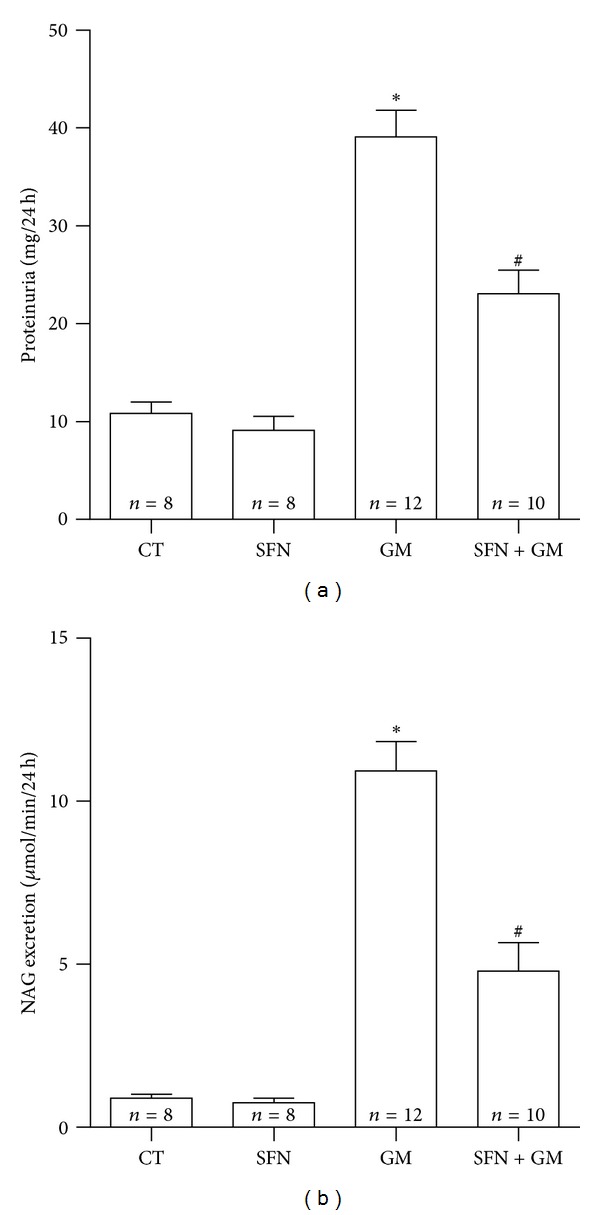
SFN ameliorates GM-induced nephrotoxicity. (a) Proteinuria and (b) urinary NAG excretion in rats. Data are mean ± SEM. *P < 0.001 versus CT and SFN; ^#^ *P* < 0.001 versus GM.

Figures 5 and 6 show the protective effect of SFN against GM-induced renal injury in rats. GM induced an increase in plasma creatinine levels (Figure 5(a)). The GM group showed increased levels of plasma creatinine (1.35 ± 0.06 mg/dL) versus CT group (0.61 ± 0.04 mg/dL). Plasma creatinine decreased in the SFN + GM group (0.91 ± 0.05 mg/dL, *P* < 0.01 versus GM). Similar trends were observed in creatinine clearance (Figure 5(b)), BUN (Figure 5(c)), and plasma GPx (Figure 5(d)). There was a decrease of just over 50% in creatinine clearance in the GM group compared to rats treated only with vehicle. SFN treatment allowed a recuperation of almost 75% of clearance capacity in the SFN + GM group compared to CT group (CT: 0.75 ± 0.02 mL/min; GM: 0.34 ± 0.03 mL/min; SFN + GM: 0.56 ± 0.04 mL/min). BUN increased in the GM group (52.5 ± 2.6 mg/dL) compared to the CT group (25.9 ± 1.8 mg/dL, *P* < 0.001) and this increase was attenuated in the SFN + GM group (40.2 ± 3.2 mg/dL, *P* < 0.01 versus GM). GPx activity showed a decrease of 1 U/mL in the GM group compared to CT group (GM: 1.26 ± 0.09 U/mL versus CT: 2.26 ± 0.11 U/mL). However, a partial reestablishment of the activity to 1.68 ± 0.07 U/mL in the SFN + GM group compared to the GM group was noted. GPx activity was similar in the CT and SFN groups. The increase of both urinary excretion of total protein (39.1 ± 2.7 mg of protein/24 h) and the lysosomal enzyme NAG (10.9 ± 0.9 *μ*mol/min/24 h) accounted for the important tubular injury induced by GM (Figures 6(a) and 6(b)). The treatment with SFN induced a remarkable attenuation of proteinuria and NAG excretion in the SFN + GM group to 23.1 ± 2.4 mg of protein/24 h and 4.8 ± 0.9 *μ*mol/min/24 h, respectively (*P* < 0.001).

### 3.5. SFN Diminishes GM-Induced Histological Alterations in Rat Renal Cortex

**Figure 7:**
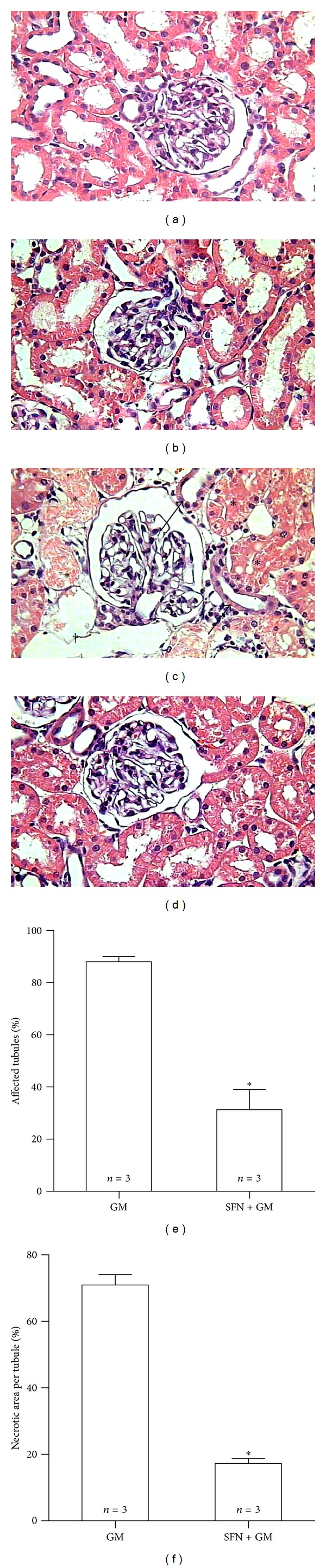
SFN attenuates GM-induced necrosis of proximal tubules. (a–d) Representative renal histopathology of rats studied (H&E staining). Sections of renal cortex with loss in brush border of proximal tubular epithelium in (c) the GM group can be observed. Asterisks represent either obstruction of tubular lumen or complete loss in tubular structure. Arrows indicate flattening of tubular walls. Cross indicates edema. These alterations are considerably diminished in (d) the SFN + GM group. (a) CT and (b) SFN groups show normal histology (40x). Quantitative morphometry shows an important decrease in (e) damaged tubules and (f) damaged area per tubule in the SFN + GM group compared to the GM group. Data are mean ± SEM. *P < 0.001 versus GM.

GM induced specific damage in the cortical convoluted tubules. This damage was variable, from mild cellular edema with loss of the apical brush border to extensive necrosis with cells detachment and luminal casts constituted by cellular debris (Figure 7). The percentage of affected tubules determined by automated morphometry in this group was of 88%, while the mean of damaged area in the convoluted proximal tubules was of 71%. SFN treatment induced a significant protection, as demonstrated by automated morphometry, which showed 31% of affected cortical tubules with 17% of damaged tubular area (Figures 7(e) and 7(f)). Kidney sections from control animals treated only with SFN or saline solution did not show any histological abnormality (Figures 7(a) and 7(b)).

### 3.6. SFN Attenuates GM-Induced Intrinsic Apoptosis in Renal Tubules and Glomeruli

**Figure 8:**
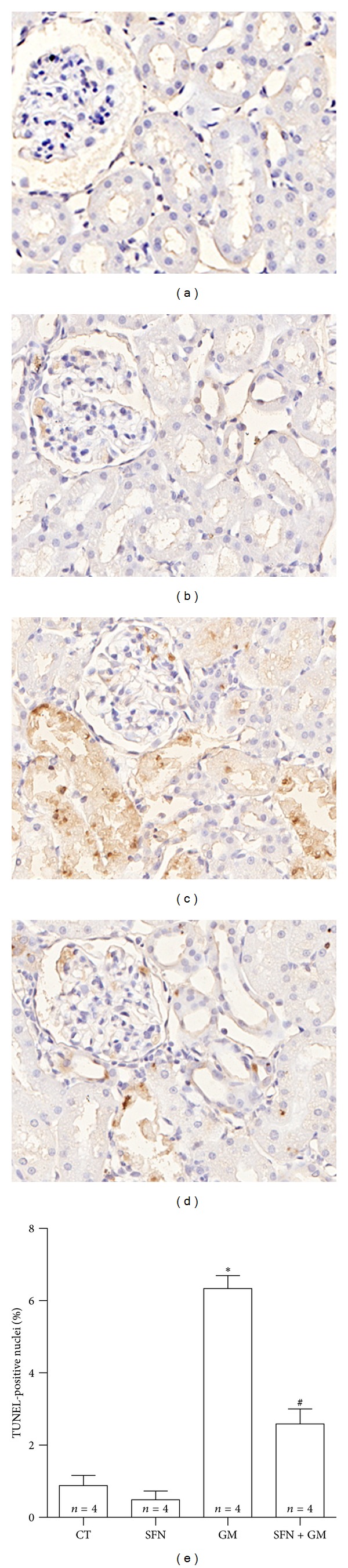
SFN attenuates GM-induced apoptosis.(a–d) Immunohistochemical detection of apoptosis by TUNEL. Sections of renal cortex of (c) rats treated with GM show nuclei intensely stained which indicate tubular cells in apoptosis. (c) There are also several nuclei of glomerular TUNEL-positive cells. Apoptosis was significantly attenuated in (d) the SFN + GM group. Basal staining was observed in renal sections from (a) CT and (b) SFN groups (40x). (e) Quantitative analysis confirms a remarkable protection of SFN against GM-induced apoptotic death in the SFN + GM group. Data are mean ± SEM. *P < 0.001 versus CT and SFN; ^#^ *P* < 0.001 versus GM.

**Figure 9:**
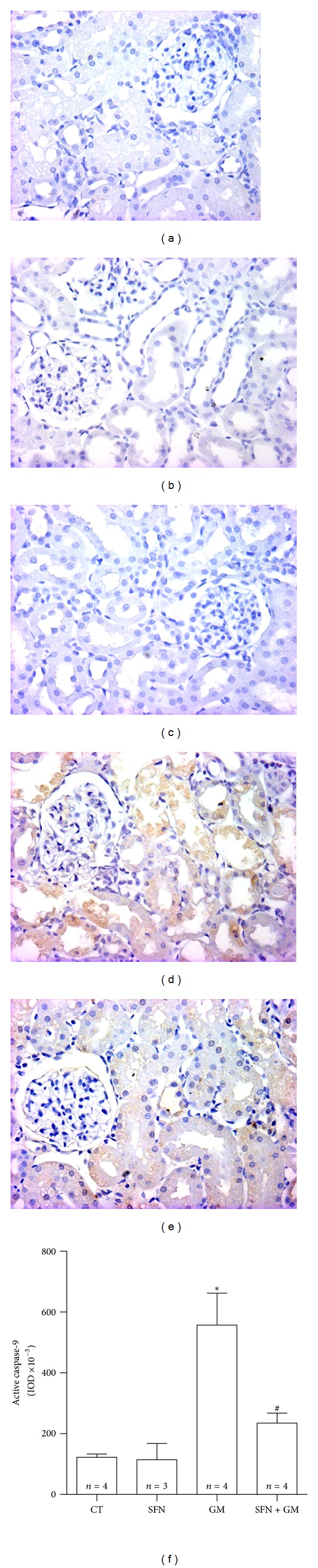
SFN prevents the GM-induced increase in active caspase-9. Representative figures are shown in (b–e) and the isotype control is shown in (a). There is an evident increase in the active caspase-9 immunostaining in (d) GM group compared to (b) CT and (c) SFN groups. The treatment with SFN in the (e) SFN + GM group reduced the caspase-9 activation (40x). (f) Quantification was made by using integrated optical density (IOD) values. Data are mean ± SEM. *P < 0.01 versus CT; ^#^ *P* < 0.05 versus GM.

Conventional histology showed numerous tubular epithelial cells with hyperchromatic condensed nuclei and conserved cytoplasm suggesting apoptosis. In order to be more specific, apoptotic cells were revealed *in situ* by TUNEL technique and quantified by automated morphometry (Figure 8). TUNEL-positive tubular and glomerular cells in kidneys of rats treated with GM are shown in Figure 8(c). Strongly stained nuclei were observed depicting early apoptosis as well as a weaker cytoplasmic staining mainly in tubules (Figure 8(c)). TUNEL positive cells were clearly decreased in the SFN + GM group (Figure 8(d)). Quantitative data, expressed as percentage of TUNEL-positive nuclei, revealed that TUNEL positivity was 7-fold higher in the GM group than in the CT group (*P* < 0.001), while the positive nuclei in the SFN + GM group were 40% of those found in the GM group (*P* < 0.001, Figure 8(e)). In order to investigate whether apoptosis was mediated by caspases as well as the apoptosis pathway involved, caspase-8 and caspase-9 were determined by immunohistochemistry. Caspase-8 was unchanged in all the studied groups (data not shown). Immunostaining of the active form of caspase-9 was observed predominately in the cytoplasm of damaged tubular cells, particularly in detached cells located in the tubular lumen and in few mesangial glomerular cells of the GM group (Figure 9(d)), being evidently higher when compared to the CT group (Figure 9(b)). The integrated optical density (IOD) was 4.6-fold higher in the GM group compared to that found in the CT group (*P* < 0.05, Figure 9(f)), while IOD measuring from the SFN + GM group was approximately 40% of the IOD determined in the GM group (*P* < 0.05) and was similar to the CT group.

### 3.7. SFN Treatment Prevents the GM-Induced Protein Nitration in Rat Renal Cortex

**Figure 10:**
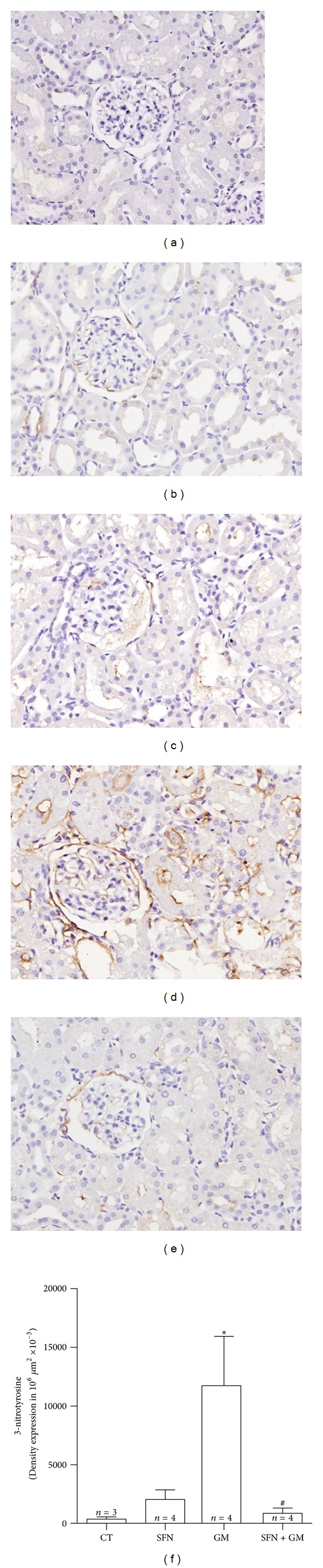
SFN treatment prevents the GM-induced protein nitration in renal cortex. Representative figures are shown in (b–e) and the isotype control is shown in (a). The immunoprevalence of 3-NT in the (d) GM group is higher when it is compared to (e) SFN + GM, (b) CT, and (c) SFN groups. This is mainly expressed in tubular cells and the parietal epithelium of Bowman (40x). (f) Quantification shows that SFN entirely prevents GM-induced protein nitration. Data are mean ± SEM. *P < 0.05 versus CT; ^#^ *P* < 0.05 versus GM.

The higher 3-NT immunostaining in the GM group (Figure 10(d)) compared to CT group (Figure 10(b)) was evident. This staining was mainly located at proximal convoluted tubules and in the parietal Bowman epithelium (Figure 10(d)). 3-NT immunostaining in the SFN + GM group (Figure 10(e)) was lower than in the GM group. Automated histomorphometry showed 31- and 13-fold higher 3-NT density expression in GM group than in the CT and SFN + GM groups, respectively (*P* < 0.05).

### 3.8. SFN Ameliorated GM-Induced Decrease in the Activity of Antioxidant Enzymes CAT and GPx in Renal Cortex

**Figure 11:**
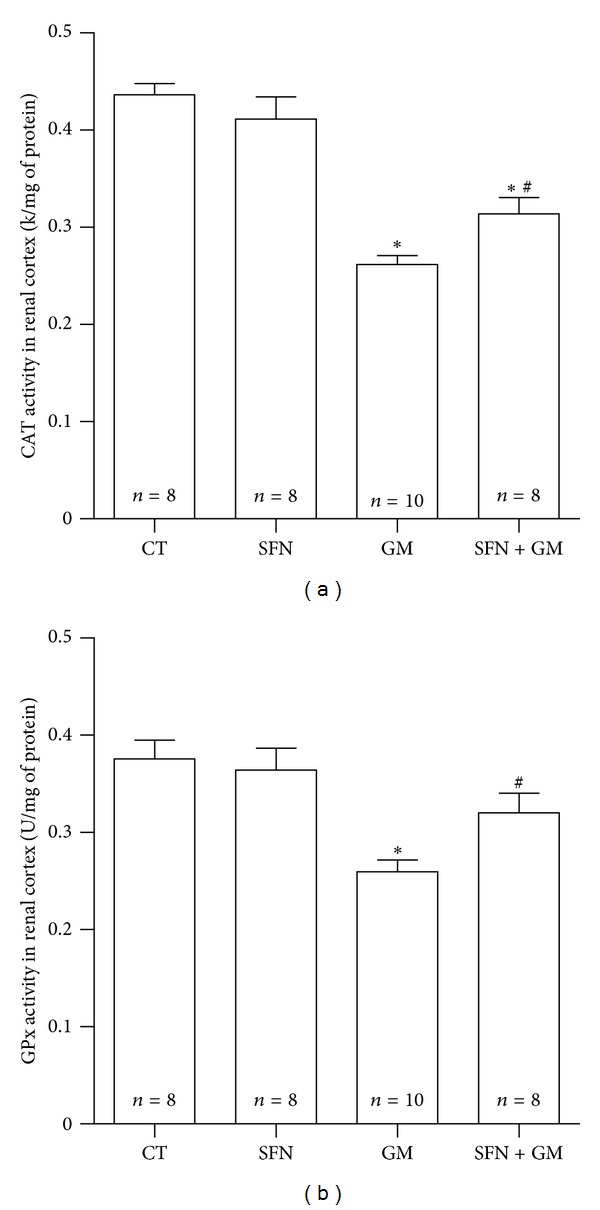
SFN attenuates GM-induced decrease in the activity of antioxidant enzymes (a) CAT and (b) GPx in renal cortex. Data are mean ± SEM. *P < 0.001 versus CT; ^#^ *P* < 0.05 versus GM.

There was a marked drop in CAT enzymatic activity in the GM group compared to the CT group (0.26 ± 0.01 versus 0.44 ± 0.01 k/mg of protein, *P* < 0.001). SFN treatment in the SFN + GM group partially reestablished the activity to approximately 71% of activity shown by the CT group (0.31 ± 0.02 k/mg of protein, *P* < 0.05 versus GM) (Figure 11(a)). A similar behavior was observed when GPx was measured (Figure 11(b)). GPx activity significantly decreased from a mean value of 0.38 ± 0.02 U/mg of protein in the CT group to 0.26 ± 0.01 U/mg of protein in the GM group (*P* < 0.001). A partial recovery of GPx activity was achieved by the SFN treatment in the SFN + GM group (0.32 ± 0.02 U/mg of protein, *P* < 0.05 versus GM).

## 4. Discussion

The *in vivo* and *in vitro* data, obtained in the present work, show that SFN is able to attenuate GM-induced nephrotoxicity. SFN was able to prevent GM-induced necrotic and apoptotic cell death. This was evident by phase contrast microscopy, MTT reduction assay, trypan blue exclusion assay, and measurement of fluorescence intensities for annexin V-FITC and PI.

On the other hand, it was found that SFN alone induces an increase (above control values of 100%) in cell viability expressed as MTT reduction but not when it is measured by direct counting of viable cells in the trypan blue exclusion assay. Our group has already observed the same behavior in LLC-PK1 cells incubated with 5 *μ*M SFN for only 24 h; in those experiments it was found that SFN prevents cisplatin-induced mitochondrial dysfunction and damage *in vitro* and *in vivo* [8]. It is known that the MTT assay is a metabolic activity assay that measures mitochondrial function and is used to detect losses in cell survive/viability due to drugs or toxicants [34]. It is possible that the observed increase in MTT reduction is associated with a promotion in mitochondrial functions and respiration rate carried out by SFN rather than an increase in cell population. The drop in MTT reduction at 10 *μ*M that forms the bell-like curve showed in Figure 1(e) is the typical hormetic response of many antioxidants.

Given the previous discussion, the protective effect of SFN against GM-induced cell death seen in this work can be linked to the SFN-induced prevention of mitochondrial dysfunction. SFN prevented the GM-induced decrease in MMP at 3 and 6 h. This fact confirms our hypothesis that SFN would prevent GM-induced cell death through mitochondrial protection. It is noteworthy that cells at 12 and 24 h of GM exposure had less green fluorescence (also less cells) indicating a seeming repolarization of mitochondria. Although there are no reports about alterations in mitochondrial dynamics induced by GM, it has been shown that mitochondria can become fused as a response to stress. Given this mechanism, damaged mitochondria can complement each other and are able to reestablish their MMP [35]. This outcome is not readily explainable but this hypothesis can be the starting point to study the effects of GM on mitochondrial dynamics.

Our *in vivo* findings corroborate the renoprotective effect of SFN. It was evident that GM-treated rats developed renal damage characterized by proteinuria, elevation in BUN and creatinine blood levels, fall in blood GPx activity and creatinine clearance, and increased urinary excretion of NAG. SFN treatment was able to reduce the GM-induced renal dysfunction, which was evident by the attenuation in the alterations of these markers in the SFN + GM group.

The precursor of SFN is contained in broccoli and this is the reason why several experiments have been done with dietary ingestion of this cruciferous vegetable [1]. The importance of the treatment with SFN instead of extracts or sprouts of broccoli in the GM-induced acute kidney injury should be mentioned. It was established that glucosinolates can be converted to isothiocyanates in humans by bowel microflora, and when this microflora is removed by mechanical cleansing and antibiotics (a mix including neomycin, an AG antibiotic), the conversion of glucosinolates became negligible [36]. Although glucoraphanin in dog and rat plasma after oral dosing of the glucosinolate was detected suggesting that a fraction of it may be absorbed intact to the plasma [37], and it was found that intact glucoraphanin can modulate carcinogen-metabolizing enzyme systems [38], it is unlikely that the broccoli consumption may be successful in this model.

Because of the intense lysosomal activity of renal tubules the raised urinary excretion of lysosomal enzymes is a sensitive marker of GM-induced tubular damage. Our findings, showing the marked decrease of urinary excretion of NAG in rats coadministered with SFN and GM, suggest an important protection of tubular structure. As expected, damaged regions shown by H&E staining were localized in the renal cortex, affecting mainly proximal tubules in kidneys of GM rats. Kidneys of SFN + GM group showed lesser tissue damage with few epithelial tubular cells affected and almost normal architecture just as the CT control.

Although typical features of necrotic death such as the edematization can be observed, the apoptotic phenotype of death was spread across the renal cortex as was revealed by the TUNEL assay. The treatment with SFN in rats of the SFN + GM group significantly attenuated the DNA fragmentation due to apoptosis. Clearly, a caspase activated in the mitochondria-dependent apoptosis pathway, caspase-9, was detected in tissues of rats from the GM group, while caspase-8, which is activated in extrinsic apoptosis, was not detected. The increment in cleaved caspase-9 was also attenuated by SFN. These last observations strengthen our findings in culture about the mitochondria-protective role of SFN in the GM-induced nephrotoxicity.

Here, it is important to highlight the known dual role of SFN. It has been reported that SFN also induces apoptosis by mitochondrial-dependent pathways in some cancer cell lines, inducing loss of MMP and activating caspase-9, at concentrations ranging from moderate (6 *μ*M) to high (25 *μ*M) [39, 40]. It seems that SFN can either prevent or induce mitochondrial damage and apoptosis in a doses-dependent way (hormesis) and/or cell-specific way, which deserves to be studied.

Immunohistochemical detection of 3-NT is a useful marker of nitrosative stress, since it has been demonstrated that this compound is formed by the reaction of reactive nitrogen species (RNS) with proteins [41]. In fact, it has been shown that nitric oxide (NO^•^) production by nitric oxide synthase—secondary to the transcriptional factor NF-*κ*B induction—is implicated in the GM-induced renal toxicity [42]. It is worth noting that NO^•^ inhibits the components of the electron transfer chain at different sensitivities. The segmental inhibition of complex I–III by NO^•^ is also followed by very high burst of superoxide production rate. As a consequence, the level of reduction of the mitochondrial components favors additional reactions of NO^•^ with ubiquinol and the formation of peroxynitrite (ONOO^−^) [43]. Interestingly, SFN was also able to entirely prevent the GM-induced elevation in 3-NT suggesting that SFN, through its indirect action, could be preventing the generation of NO^•^, the mitochondrial dysfunction mediated by NO^•^, and the formation of ONOO^−^ involved in protein nitration.

Finally, it was found that SFN attenuated the loss of activity of the antioxidant enzymes catalase and GPx in renal cortex. It has been demonstrated that catalase is affected by ROS due to its high susceptibility to carbonylation [44], and GPx can be inactivated by NO^•^, carbonylic compounds, or diminished GSH levels [45, 46]. Given those facts, probably the renoprotective effect of SFN is also mediated by controlling the generation of ROS/RNS and prevention of oxidative/nitrosative stress.

In conclusion, our results show that SFN treatment protects kidneys and renal tubular cells against GM-induced toxicity and suggest that this protective effect would be associated with prevention of nitrosative stress and/or, mainly, preservation in mitochondrial functions.

## Figures and Tables

**Table 1:** Counting of daily detached, trypan blue stained and viable LLC-PK1 cells during the GM exposure and SFN treatment.

	CT	SFN	GM	SFN + GM
DC/well 24 h	1,483 ± 367	483 ± 60	5,487 ± 247*	1,113 ± 567^#^
DC/well 48 h	1,633 ± 109	1,117 ± 469	8,033 ± 324*	4,250 ± 375^#^
DC/well 72 h	2,867 ± 192	2,883 ± 259	8,017 ± 760^*ε*^	4,733 ± 838^Δ^
TBS cells/well (attached after GM exposure)	16,517 ± 3,219	23,733 ± 8,020	31,800 ± 3,318	25,433 ± 829
Total death cells/well	22,500 ± 3,399	28217 ± 8,366	53,337 ± 3,714^*ε*^	35,550 ± 1,422
Viable cells/well	106,450 ± 4,976	94,317 ± 5,874	26,883 ± 4,681*	60,400 ± 5,765^Δ^

CT: cells without treatment. SFN: cells incubated in 5 *µ*M SFN. GM: cells in 8 mM GM. SFN + GM: cells coincubated in 5 *µ*M SFN and 8 mM GM. DC: detached cells. TBS: trypan blue stained. Total death cells = DC 24 h + DC 48 h + DC 72 h + TBS cells. Cells attached after GM exposure include TBS cells + viable cells. Data are mean ± SEM, *n* = 3. *P < 0.001 versus CT; ^#^ *P* < 0.001 versus GM; ^*ε*^ *P* < 0.01 versus CT; ^Δ^ *P* < 0.05 versus GM.

**Table 2:** Urine volume, body weight, right kidney weight, and right kidney ratio at the end of treatment in all the studied groups.

	CT	SFN	GM	SFN + GM
Urine volume (mL/day/100 g BW)	5.9 ± 0.5	5.7 ± 1.4	8.4 ± 0.9	6.7 ± 1.1
Body weight (g)	238.9 ± 5.1	227.0 ± 4.4	226.0 ± 4.0	222.2 ± 3.4
Right kidney weight (g)	0.89 ± 0.02	0.91 ± 0.03	1.13 ± 0.04*	1.12 ± 0.03*
Right kidney ratio (%)	0.37 ± 0.01	0.40 ± 0.01	0.50 ± 0.01*	0.50 ± 0.01*

BW: body weight. Kidney ratio = (kidney weight/body weight) × 100. Data are mean ± SEM, *n* = 8–12. *P < 0.001 versus CT.
